# A Comprehensive Review of Blockchain Technology-Enabled Smart Manufacturing: A Framework, Challenges and Future Research Directions

**DOI:** 10.3390/s23010155

**Published:** 2022-12-23

**Authors:** Xin Guo, Geng Zhang, Yingfeng Zhang

**Affiliations:** School of Mechanical Engineering, Northwestern Polytechnical University, Xi’an 710072, China

**Keywords:** blockchain, data sharing, data security, trust mechanism, smart manufacturing

## Abstract

As a new generation of information technology, blockchain plays an important role in business and industrial innovation. The employment of blockchain technologies in industry has increased transparency, security and traceability, improved efficiency, and reduced costs of production activities. Many studies on blockchain technology-enabled system construction and performance optimization in Industry 4.0 have been carried out. However, blockchain technology and smart manufacturing have been individually researched in academia and industry, according to the literature. This survey aims to summarize the existing research to provide theoretical foundations for applying blockchain technology to smart manufacturing, thus creating a more reliable and authentic smart manufacturing system. In this regard, the literature related to four types of critical issues in smart manufacturing is introduced: data security, data sharing, trust mechanisms and system coordination issues. The corresponding blockchain solutions were reviewed and analyzed. Based on the insights obtained from the above analysis, a reference framework for blockchain technology-enabled smart manufacturing systems is put forward. The challenges and future research directions are also discussed to provide potential guides for achieving better utilization of this technology in smart manufacturing.

## 1. Introduction

With the deep integration of information technologies (e.g., artificial intelligence, cloud computing, big data) and the manufacturing industry, smart manufacturing has been recognized as one of the main driving forces of Industry 4.0 [1]. Smart manufacturing aims to improve product and service quality, increase efficiency and promote innovative, green and coordinated manufacturing, which has increasingly become a major trend of the manufacturing industry and an essential motivation for enhancing manufacturing enterprises’ core competitiveness [2]. Although there is a growing concern about the importance of smart manufacturing, there are still a number of problems and challenges in achieving smart manufacturing, such as data security risks, information asymmetry, trust mechanism deficiency, the lack of system coordination, etc. [3,4]. These issues seriously restrict the development of smart manufacturing and urgently need to be solved by innovative methods and technologies.

Since Satoshi Nakamoto first put forward the concept of blockchain as a platform for bitcoin transactions [5], it has attracted wide attention from academia and industry. Blockchain can be regarded as a distributed database of records or shared public/private ledgers that have been executed and shared among different participating parties [6]. Four critical technical properties are included in the blockchain, namely decentralized structure, cryptography system, consensus mechanism and smart contract [7,8]. With these properties, blockchain technology can guarantee the reliability, traceability and authenticity of information. Moreover, smart contractual relationships help create a trusted environment for smart manufacturing [9,10,11]. Due to its promising characteristics, blockchain is supposed to be the key enabling technology for reorganizing huge amounts of data and intelligent agents scattered in the manufacturing system. In general, with the adoption of blockchain technology, a more socialized smart manufacturing system is constructed by connecting intelligent agents and making them credible, reliable and cooperatively working together [12].

As discussed above, by integrating encryption algorithms, consensus mechanisms, smart contracts and other technologies, blockchain serves as a potential facilitator of data authenticity, privacy protection, information traceability and performance optimization [13]. The specific properties of blockchains show promising application prospects in smart manufacturing. The current status of blockchain applications in manufacturing systems and how blockchain systems can overcome potential cybersecurity barriers to achieving intelligence in Industry 4.0 was discussed by Leng [3]. A systematic literature review and explained the potential contributions of blockchain technology to the economic, environmental and social performances of manufacturers and their supply chains was conducted by Khanfar [14]. A systematic review of blockchain applications to intelligent transportation systems in general and the Internet of Vehicles (IoV) in particular was provided by Jabbar [15]. However, the current research did not fully reveal the advantages of applying blockchain technology to smart manufacturing. Therefore, this survey explores the potential implementation of blockchain technology in smart manufacturing systems to enhance its authenticity, reliability and overall performance. To achieve this, current critical issues in smart manufacturing systems and the corresponding blockchain solutions are discussed to help develop a deep understanding of applying blockchain technology in smart manufacturing. This study also reveals how these critical issues have been studied in the literature. Based on this, we put forward a reference framework for blockchain technology-enabled smart manufacturing systems. Finally, the current challenges and future research directions for applying blockchain technology in smart manufacturing are analyzed.

The rest of the paper is organized as follows. Section 2 introduces the research method. After reviewing current critical issues in smart manufacturing systems in Section 3, Section 4 discusses the key technologies of blockchain and its advantages in smart manufacturing systems. A review of how these critical issues have been studied in the literature is further explored in Section 5. The proposed reference framework for blockchain technology in smart manufacturing is illustrated in Section 6. Challenges and future research directions are discussed in Section 7. A conclusion on how to use blockchain technology to support smart manufacturing is drawn in Section 8. See the end of the document for further details on the references.

## 2. Literature Search

A literature search was conducted in the Web of Science database, where a broad range of literature on blockchain technology-enabled smart manufacturing can be identified. Articles retrieved were further searched using a three-step approach. 

The first step was to obtain quality publications through certain selection criteria. Working papers and commentaries were excluded to derive quality publications. Meanwhile, the keywords “blockchain” and “smart manufacturing” were identified for searching publications. A total of 138 publications were obtained and used for further analysis (up to 21 September 2021).

The second step was theoretical screening. Articles about blockchain technology and its application in the manufacturing industry were included to help develop a deep understanding of blockchain technology-enabled smart manufacturing. To be specified, the selection criteria are shown as follows.

Blockchain applications manufacturing in industry were selected, including applying blockchain to supply chain management, manufacturing management and Internet of Things (IoT). These studies focused on the industry, product or service, and governance cases of blockchain implementations. Also, reviews on blockchain and its relevant technologies were investigated to provide a comprehensive overview of research trends, technologies and issues. These articles will help identify the challenges of implementing blockchain technology in smart manufacturing. 

The exclusion criteria were (1) research not related to the blockchain domain or manufacturing management; (2) papers that were not peer-reviewed or less than four pages; and (3) papers not written in English.

In the last step, Reference Analysis, 87 articles that met the selection criteria were selected, and the cited references were further utilized as a source for literary analysis, resulting in the identification of 7 additional articles. Additionally, 8 supplementary references were added to the reference section to make the survey concrete. Therefore, this survey consisted of 102 articles in total. The following two sections aim to discuss and analyze blockchain applications in the smart manufacturing industry. Four critical issues have been identified in the area of smart manufacturing. How blockchain technology can overcome these critical issues to support smart manufacturing was also investigated. 

## 3. Critical Issues in Smart Manufacturing

With the rapid development of advanced information technologies, such as the IoT, cyber physical systems, artificial intelligence and cloud/edge computing, the manufacturing industry has entered a new stage called smart manufacturing [16,17]. The efficiency of manufacturing has been greatly improved and the manufacturing processes have been constantly optimized. However, at present, smart manufacturing still faces problems in many aspects, such as data security risks, information asymmetry, trust mechanism deficiency and the lack of system coordination. Novel information technologies urgently need to be adopted to solve these problems. The following section discusses the existing critical issues faced by smart manufacturing.

### 3.1. Data Security Issues

Various advanced information technologies are applied in smart manufacturing to connect manufacturing processes with Internet information systems, resulting in severe challenges to data and information security [18]. At present, key data from enterprises exposed to the IoT are facing the danger of leakage and illegal usage. Enterprises’ production management systems, office networks and other key information infrastructures are undergoing serious threats of network attacks, virus invasion and so on [19,20]. Although many companies have realized the risks of data transformation, most of them excessively depend on traditional protection mechanisms [21], such as Role-Based Access Control (RBAC), Veeam Endpoint Backup and Storage Disaster Recovery Service. Due to the lack of effective information security methods, the unpredictable and inevitable network attack eventually brings serious economic losses to smart manufacturing enterprises.

### 3.2. Data Sharing Issues

Effective data sharing can improve productivity, reduce cost and promote the overall development of smart manufacturing enterprises [22]. It should be noted that there are still many problems with manufacturing data sharing. First, the enterprises’ industrial sectors lack the motivation to public relevant data and information due to their concern for self-interests. Second, manufacturing data sharing is often implemented by the isolated information systems of different enterprises. The heterogeneous databases and diverse standards applied by different systems increase the difficulty of data integration across systems [23]. Finally, the potential value of manufacturing data could hardly be extracted and effectively utilized by smart manufacturing enterprises due to the lack of an advanced information management approach.

### 3.3. Trust Mechanism Issues

The rapid development of the smart manufacturing industry inevitably brings about serious trust issues. First, the information asymmetry leads to distrust between various smart manufacturing enterprises and restricts the effective collaboration of the production chain. Second, due to the potential risk of manufacturing data leakage, tampering and disclosure, it is relatively difficult to trace the abnormal products and corresponding information during the manufacturing process. The development of trusting relationships among related enterprises is obstructed because of incomplete information traceability mechanisms [24]. Finally, the authenticity of manufacturing data cannot be strictly guaranteed, resulting in the potential risk of product adulteration during the process of production and sales, which eventually causes serious damage to the credibility and reputation of enterprises [1].

### 3.4. System Coordination Issues

At present, the lack of standardization and innovation of enterprises and the imperfection of supporting systems greatly obstruct the overall development of smart manufacturing [25]. On the one hand, many restrictive factors seriously impede the cooperative process of the smart manufacturing industry, such as the lack of independent intellectual property rights, the weakness of research and development capability, and the uncertainty of manufacturing and its management [26,27]. On the other hand, from the perspective of enterprises, many of them are still at the stage of the traditional manufacturing mode and are not willing to actively participate in the revolution toward smart manufacturing and tend to make independent decisions based on their own principles and arrangements. The individuation of enterprises objectively obstructs the collaborative development of the smart manufacturing industry [28].

## 4. Blockchain and Its Advantages in the Manufacturing System

As a newly emerged revolutionary information technology with the rapid development of digital cryptocurrencies, such as Bitcoin, blockchain is essentially a decentralized distributed database that allows peer-to-peer transactions without a third party. With its encryption algorithm, consensus algorithm, smart contract and other technologies, blockchain can be adopted to promote security protection, information traceability, data authenticity and system collaboration [29]. It has gradually penetrated various industries and formed exploratory applications in many fields. This section discusses the important role of blockchain technology in smart manufacturing.

### 4.1. Key Technologies of Blockchain

**Figure 1:**
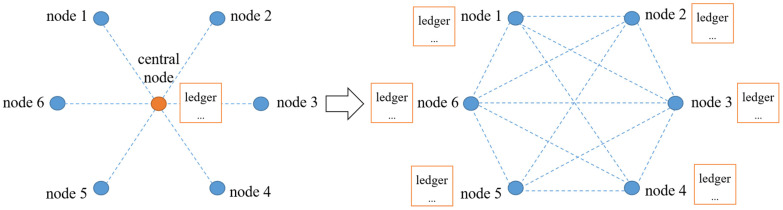
Comparison of traditional storage and distributed storage.

**Figure 2:**
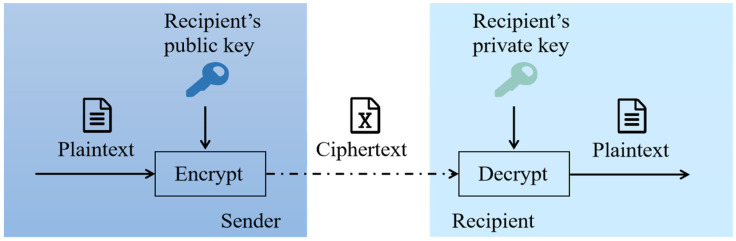
Asymmetric key encryption process.

**Figure 3:**
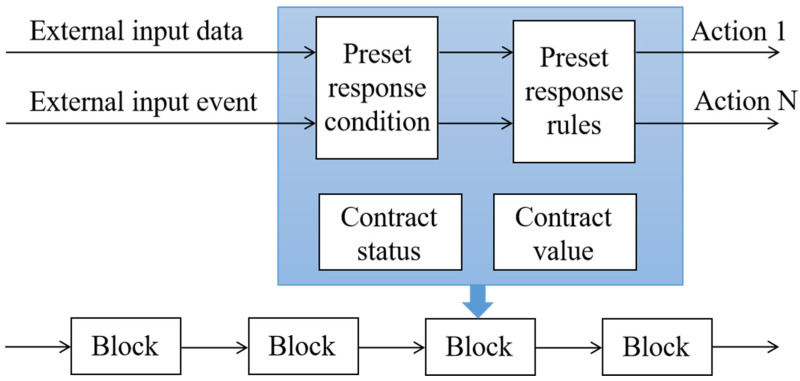
The operation mechanism model of a smart contract.

(1) Distributed ledger: Distributed ledger technology (DLT) is essentially decentralized data storage technology that realizes data sharing and synchronization based on a network composed of multiple nodes [30,31,32]. The traditional storage system implements a data management mechanism controlled by a central node or authority, while the DLT adopts a multi-party decision-making and joint maintenance method based on a consensus mechanism, as shown in Figure 1. The decentralized data maintenance strategy of a distributed ledger can effectively reduce system maintenance costs and improve system efficiency [33,34].

(2) Asymmetric encryption: Asymmetric encryption technology, also known as Public Key Infrastructure (PKI), usually uses two asymmetric passwords in the process of encryption and decryption, which are called public keys and private keys [35,36]. Asymmetric key pairs have two distinguished characteristics. First, after encrypting information with one key (public key or private key), only the other corresponding key can be used for decryption. Second, the public key can be disclosed to others, while the private key is confidential. The encryption process is illustrated in Figure 2. At present, common asymmetric encryption algorithms are hash function, RSA, Elgamal, D-H and elliptic curve encryption algorithm (ECC) [37]. Blockchain technology mainly utilizes ECC, which has high security performance and low complexity.

(3) Consensus mechanism: A consensus mechanism is a series of common rules for each decentralized node to be consistent. It enables each node to verify and confirm the data in the network so as to maintain the fairness of the whole system and enhance trust among different participants [38]. As listed in Table 1, common consensus mechanisms include Proof of Work (PoW), Proof of Stake (PoS), Delegated Proof of Stake (DPoS) and Practical Byzantine Fault Tolerance (PBFT) [39,40,41]. Taking bitcoin as an example, with the adoption of PoW, it is possible to forge a non-existent record only when more than 51% of the accounting nodes in the whole network reach agreement, which is basically impossible when a large number of nodes join the blockchain, thus eliminating the possibility of counterfeiting.

(4) Smart contract: Smart contract is a computer protocol designed to propagate, validate, or execute contracts in a virtualized manner [42,43]. In essence, it is an executable code written into the blockchain network. Once an event triggers the terms in the contract, the code is executed automatically [44,45]. The operation mechanism of the smart contract is shown in Figure 3. The application of smart contracts in blockchain networks facilitates trusted transactions without a third party, reduces transaction costs and improves transaction efficiency [46].

### 4.2. Blockchain Solutions to Critical Issues in Smart Manufacturing

(1) Data security: Blockchain ensures data security in a smart manufacturing system based on its chain storage structure with a timestamp [47,48,49]. Each block of blockchain contains a timestamp and a link to the previous block. Once a piece of data is recorded in a block, it cannot be tampered with. The encryption algorithm guarantees data security [50]. In addition, the traceability of blockchain also facilitates the optimization of manufacturing systems [51,52]. In a real scenario, blockchain technology-enabled Industrial Internet of Things (IIoT) can effectively prevent information leakage and malicious manipulation risks brought by malicious attacks on any single node device.

(2) Data sharing: With the adoption of DLT, blockchain simultaneously allows information recording and sharing by all participants [53]. This helps realize real-time data sharing between upstream and downstream enterprises. At the same time, due to its privacy protection mechanism, confidential information is encrypted to solve the contradictions between data privacy and data sharing [54]. Therefore, blockchain technology can eliminate information isolation and realize effective data sharing between different parties. In a real scenario, a logistics data-sharing platform based on blockchain technology can speed up document transfer and account reconciliation, thus improving overall logistics efficiency.

(3) Trust mechanism: Blockchain technology, known as a “trust machine,” can provide trust services for all parties in smart manufacturing [55]. The key data during the process of design, production and sale are collectively maintained based on trusted mechanisms [56,57]. Manufacturers, suppliers, distributors and other smart manufacturing entities share transparent and credible information. Trust relationships are, therefore, established among different parties [58,59]. In real scenarios, supplier background investigation and product quality inspection can be eliminated with the application of blockchain technology, further reducing the cost of smart manufacturing.

(4) System coordination: Paperless automatic transactions and trustworthy electronic storage based on blockchain greatly improve the transaction efficiency of enterprises [60,61,62]. Moreover, the smart contract of blockchain technology promotes collaboration between upstream and downstream enterprises in the smart manufacturing industry chain. As a result, it becomes an effective driving force for the coordinated development of the whole smart manufacturing system [63,64,65,66]. In a real scenario, a smart procurement platform based on blockchain can promote procurement coordination and improve the transparency of trade relations within the industry.

## 5. Application of Blockchain Technology in Smart Manufacturing

As discussed in Section 3, four types of critical issues of manufacturing systems are overviewed, including data security issue (C1), data sharing issue (C2), trust mechanism issue (C3) and system coordination issue (C4). Table 2 offers an overview of the research on the application of blockchain technologies in smart manufacturing with regard to their addressed issues.

### 5.1. Research on Data Sharing and Data Security in Smart Manufacturing

From the perspective of data sharing and data security, many scholars have explored blockchain to promote data sharing and data security with regards to its features of immutability, transparency, automation and integrity. A multi-center, partially decentralized IIoT architecture to enhance security and privacy based on blockchain technology was introduced by Wan [67]. The integrated methods of blockchain and machine learning to solve data security and management issues in smart manufacturing was proposed by Shahbazi and Byun [68]. Blockchain technology was utilized to improve the privacy and security of data transmission and communications in the Internet of Things (IoT) by Zhu and Souri [69]. A generalized architecture applying blockchain technology in smart agriculture to provide security goals was proposed by Vangala [70]. An intelligent manufacturing security model supported by the blockchain with the purpose of enhancing security, privacy and non-tamperability was proposed by Xu [71]. A use case of blockchain proposed framework for avoiding fraud scenarios and securing logistics trade was demonstrated by Abhishek [72].

### 5.2. Research on Traceability and Trust Mechanisms in Smart Manufacturing

From the perspective of traceability and trust mechanisms, many scholars have discussed the application of blockchain technology to ensure the authenticity and credibility of data and to enhance trust among smart manufacturing enterprises. Blockchain-based trust mechanism for quality assurance aiming to promote the transparency, security and efficiency of transactions was proposed by Zhang [56]. Blockchain technology was applied to enhance the security of data in smart factories by Kho and Jeong [73]. A ubiquitous manufacturing platform based on blockchain to provide a peer-to-peer communication network between the end user and the service provider was proposed by Barenji [74]. A manufacturing system in a pharmaceutical environment taking advantage of blockchain properties and smart contracts to ensure data authenticity, transparency and immutability was proposed by Leal [75]. Blockchain technology was applied in a cloud manufacturing system in order to solve the trust problem and resource scheduling efficiency problem by Cheng [76]. A blockchain platform to maintain a decentralized medical supply chain and promote the traceability of the overall system was proposed by Panda and Satapathy [77].

### 5.3. Research on System Construction and Performance Optimization in Smart Manufacturing

From the perspective of system construction and performance optimization, many scholars have acknowledged blockchain as a significant approach to enhance collaboration among smart manufacturing enterprises and promote the cooperative development of the smart manufacturing industry. As for using blockchain technology for system construction, private-blockchain-based IIoT to facilitate the exchange of product and material tracking information while ensuring confidentiality was proposed by Assaqty [24]. A blockchain-based platform for Industry, named BPIIoT, to process all transactions based on blockchain networks was proposed by Bai [81]. The Blockchain-based Shared Manufacturing (BSM) framework to support the application of Cyber Physical Systems (CPS) was proposed by Yu [83]. A novel hybrid intelligence model, ManuChain, which takes advantage of blockchain-driven smart contracts to proactively decentralize task execution and make the results available for optimization was proposed by Leng [61]. As for using blockchain technology for performance optimization. A smart manufacturing system applying blockchain technology to promote both device-level data transmission and manufacturing service transactions was proposed by Lee [78]. Research on blockchain reference system architecture, aiming to promote applicability and consistency across the enterprise infrastructure was conducted by Yalcinkaya [79]. Blockchain technologies and smart contracts was applied to address trust issues between cloud service providers (CSP) and cloud service consumers (CSC) while ensuring the effectiveness and efficiency of business services by Tan [80]. A smart manufacturing conceptual scenario with the integration of blockchain to strengthen data integrity and decrease data transmission risk was proposed by Shahbazi and Byun [82]. Blockchain technology was used to secure and improve data trust to avoid fake data during system transactions by Shahbazi and Byun [84]. 

## 6. Reference Framework of Blockchain Technology-Enabled Smart Manufacturing

**Figure 4:**
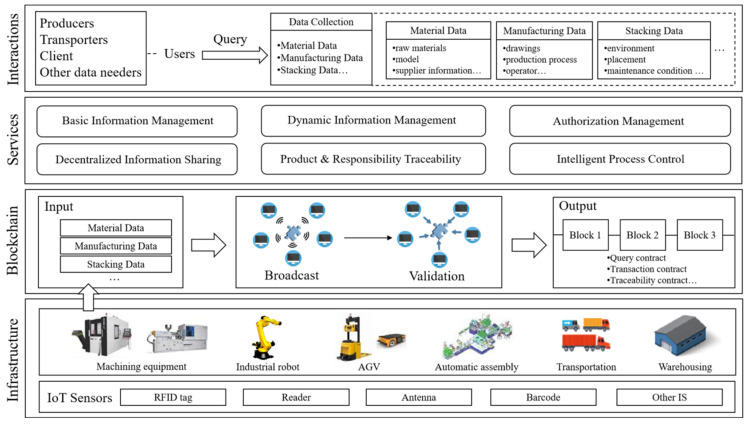
Reference framework of blockchain technology-enabled smart manufacturing.

The reference framework for blockchain technology-enabled smart manufacturing is presented in Figure 4. The framework mainly contains four layers, namely the infrastructure layer, blockchain layer, services layer and interaction layer. 

The infrastructure layer is responsible for recording, collecting, temporarily storing and transferring data. This is the basis of the proposed framework. The blockchain layer, as the core of the whole system, stores all the required information for service operations. The main task of the service layer is to process and manage massive manufacturing data. The interaction layer allows authorized users to query all sorts of manufacturing information and to promotes information sharing. 

To ensure the accuracy of data collection, the proposed framework is constructed based on IoT technology, which significantly reduces deficiencies caused by artificial constraints, such as time delays and data errors [65]. All physical objects are equipped with electronic IoT-sensing equipment so that data can be automatically collected without manual operations [85]. A series of smart manufacturing information, such as those relating to manufacturing, assembly, transportation and warehousing, are sequentially acquired by IoT sensors and transferred to the blockchain layer.

The blockchain layer is responsible for processing and storing the information uploaded by the infrastructure layer. The input data include material data, manufacture data, transport data, stacking data, etc. The data can be divided into two types: data collected from intelligent sensing equipment and data uploaded by authorized relevant parties. All sort of data from procurement, manufacturing and transportation processes will be transferred to the blockchain. The storage process was conducted as follows. First, the input data are packaged into the candidate block and then broadcast to the entire network. After that, the validation process is conducted based on a consensus mechanism. If the candidate block is verified by most nodes, it is stored directly in the blockchain. 

The services layer provides six services, namely information management, authorization management, product traceability and process control. These services can further facilitate the interaction layer in performing accurate and efficient information querying. In general, information management can be divided into two types: basic and dynamic information management. Basic information management is mainly used for input data that do not change regularly in smart manufacturing, such as product size, performance and materials. Dynamic information management is applied to information that frequently changes due to uncertain external conditions, such as logistics and stacking information. Authorization management is provided for user’s identity information authentication before accessing data stored in the blockchain. Decentralized information sharing realizes real-time data sharing between the upstream and downstream enterprises of the smart manufacturing industry chain. Product & responsibility traceability utilizes smart contracts to generate unique trace codes for tracing the whole process product information. Intelligent process management helps analyze, predict and optimize the smart manufacturing process based on data stored in the blockchain.

The interaction layer implements an information query. Relevant parties to smart manufacturing can acquire the needed information using their unique digital identity. By accessing the system, a dataset containing specific smart manufacturing information can be obtained by relevant parties.

## 7. Challenges and Future Research

As a revolutionary new technology, blockchain significantly improves the efficiency, security and trustworthiness of smart manufacturing systems. However, the theoretical research and industrial practices of blockchain technology are still at an exploratory stage. Many challenging issues need to be overcome before the benefits of blockchain can be fully realized. In this section, we focus on the current challenges and future research on blockchain technology-enabled smart manufacturing systems. We believe that it is essential to investigate the potential contributions of blockchain technologies to smart manufacturing in different sectors and promote research on the implications of blockchain technology for smart manufacturing. Specifically, challenges and future research are listed in the following three aspects:

(1) System integration

First, most IoT devices have poor computing power and limited storage space. However, the decentralized consensus algorithm of blockchain tends to require significant computing power and energy consumption. For example, the PoW algorithm for Bitcoin consumes high energy [86]. Therefore, consensus mechanisms with large power consumption may not be feasible for low-power IoT devices. The large amount of blockchain data also makes it impossible to fully deploy on smart manufacturing systems. In the future, the integration of edge computing and cloud computing technology into blockchain may overcome the resource limitation of Internet of things devices. 

Second, applying blockchain technology to manufacturing information systems requires data synchronization and transformation from the current enterprise information systems. As blockchain holds continuously growing transaction records, there exists the architectural design issue of effectively splitting a large amount of transaction data between the operational level and the enterprise level [87]. With a split blockchain, a lighter operational blockchain of higher performance is available at the operational level [88,89]. Meanwhile, a complete history of transactions is still available at the enterprise level. Further research is needed to make a smooth transition from a traditional central system to a blockchain-based information system.

(2) Privacy protection

First, the adoption of blockchain technology in smart manufacturing can improve its security through asymmetric encryption and digital signatures, but there are still risks of cyberattacks due to the vulnerability of blockchain systems. For example, Malicious users can hijack blockchain messages using a border gateway protocol (BGP) routing scheme, resulting in higher block broadcast latency [90]. The peer-to-peer (P2P) network of blockchain may also bring security issues, as data transfer and consensus processes are all performed based on P2P networks. P2P systems may be subject to various networking attacks, such as DoS, ARP spoofing, Sybil and routing attacks [91,92]. System vulnerability must be seriously considered before blockchain technology can be fully applied to smart manufacturing.

Second, blockchain technology ensures the data privacy of transactions to a certain extent. Bitcoin, for example, is conducted through Internet protocol (IP) addresses rather than users’ real identities, thus ensuring anonymity. In addition, single-use accounts are created in Bitcoin to ensure user anonymity. However, these conservation mechanisms are still not robust enough. A user’s pseudonym can be cracked by learning and deducing multiple transactions related to a common user [93]. The complete storage of transaction data on the blockchain may also lead to potential privacy breaches and a memory optimization and flexible blockchain data storage scheme may reduce the risk of privacy leakage to some extent [94]. Future work is needed to develop frameworks for improving the security and privacy of blockchain technology-enabled smart manufacturing.

(3) System scalability

First, the system throughput of blockchain is very limited. It can only handle small volumes of transactions. For example, Bitcoin can only process 7 transactions per second [95,96]. While VISA’s credit card platform can process about 2000 transactions per second, PayPal’s payment platform can process 170 transactions per second [97]. In short, existing blockchain systems may not be suitable for high-volume applications. To address the low throughput of blockchain, one direction is to develop a more efficient consensus algorithm. A new distributed protocol to improve the throughput of PoW, which first solved difficult problems with a small computational load and then reached consensus in multiple groups was proposed by Luu [98]. Future research should focus on improving blockchain throughput while maintaining its characteristics of data transparency, immutability and trustworthiness.

Second, the scalability of existing blockchain systems limits their widespread use in large-scale smart manufacturing. The system could be expanded to include numerous participants, such as IIoT smart devices, suppliers, manufacturer users, cloud and edge servers. As the number of participants and the size of the ledger increase, the amount of data being processed and stored in the chain keeps growing. As a result, system latency and storage costs gradually increase, while throughput decreases. In general, the performance of a blockchain system decreases as the number of users and devices increases [99,100]. The scalability issues could be addressed by three schemes, including off-chain payment network, Bitcoin-NG and sharding mechanism [101,102]. Future work should be devoted to solving the blockchain scalability issue and creating a high-performance blockchain technology-enabled smart manufacturing system.

## 8. Conclusions

Many studies on blockchain technology and smart manufacturing in Industry 4.0 have been conducted individually, but the research on simultaneously applying blockchain technology in smart manufacturing is still in its infancy. To address this limitation, the article surveys the research progress of blockchain technology-enabled smart manufacturing and provides insights for future research in this field. The following contributions were made by this review:

First, a comprehensive review of blockchain technology in smart manufacturing was conducted. The concepts of blockchain technology and its key technologies are characterized in detail. Four types of critical issues in smart manufacturing were identified and the corresponding blockchain solutions were discussed. The potential applications and advantages of applying blockchain technology in smart manufacturing were summarized through a literature review.

Second, a conceptual framework for blockchain technology-enabled smart manufacturing systems was proposed. The framework can be used as a guideline to help develop a deep understanding of how to integrate blockchain into various smart manufacturing applications.

Finally, current challenges and future research related to blockchain as applied to smart manufacturing are discussed. Potential solutions for future improvement were also provided. Future work should focus on ways to improve system integration, privacy protection and system scalability.

## Figures and Tables

**Table 1:** Comparison of common consensus mechanisms.

	PoW	PoS	DPoS	PBFT
Application	Bitcoin, Ethereum, LiteCoin, Dogecoin	Ethereum, Peercoin, Nxt	BitShares, Steemit, EOS, Lisk, Ark	Hyperledger Fabric, Stellar, Ripple, Dispatch
Classification	Competitive consensus	Competitive consensus	Collaborative consensus	Collaborative consensus
Advantages	Easy to implement, high security and difficult to attack	Low computing resource consumption, high efficiency	High throughput, fast operation speed	High-speed and scalable
Disadvantages	Huge energy consumption; low operating efficiency	Complex protocol, high network requirements	Slightly centralized, easy to cause collusion attacks	Only used for private and consortium blockchain

**Table 2:** Research on the application of blockchain technologies in smart manufacturing.

Function	Literature	Addressed Issues	Details
Data sharing and data security	[67]	C1, C2	The proposed multi-center partially decentralized IIoT architecture uses blockchain to enhance security and privacy
	[68]	C1	The study applies the integrated methods of blockchain and machine learning to solve data security and management issues in smart manufacturing
	[69]	C1, C2	The utilized blockchain technology to improve the privacy and security of data transmission and communications in IoT
	[70]	C1	The proposed generalized architecture applies blockchain technology in smart agriculture to provide security goals
	[71]	C1, C2, C3	The proposed intelligent manufacturing security model supported by the blockchain can effectively enhance security, privacy and non-tamperability
	[72]	C1, C2	The proposed use case of a blockchain framework is aim to avoid fraud scenarios and secure logistics trade
Traceability and trust mechanism	[56]	C1, C2, C3	The blockchain-based trust mechanism for quality assurance promotes the transparency, security and efficiency of transactions
	[73]	C1	The study applies blockchain technology to enhance the security of data in smart factories
	[74]	C1, C2, C3	The proposed platform uses blockchain to provide a peer-to-peer communication network between the end user and the service provider
	[75]	C1, C2, C3	The proposed system in a pharmaceutical environment takes advantage of blockchain properties and smart contracts to ensure data authenticity, transparency and immutability
	[76]	C3, C4	The study utilizes blockchain technology to solve the trust problem and resource scheduling efficiency problem in a cloud manufacturing system
	[77]	C1, C3	The study applies a blockchain platform to maintain a decentralized medical supply chain and promote the traceability of the overall system
System construction and performance optimization	[78]	C1, C2	The proposed system utilizes blockchain technology to promote both device-level data transmission and manufacturing service transaction
	[79]	C1, C2	The proposed blockchain reference system architecture promotes applicability and consistency across enterprise infrastructure
	[80]	C2, C3	The study applies blockchain technologies and smart contracts to address trust issues while ensuring the effectiveness and efficiency of business services
	[24]	C1, C2	The proposed private-blockchain-based IIoT is aimed to bridge the need for product and material tracking information exchange while ensuring confidentiality
	[81]	C1, C2	The proposed blockchain-based platform for Industrial Internet of Things (BPIIoT) applies blockchain network to process all transactions, including digital signature and programmable permission
	[82]	C1, C2	The proposed smart manufacturing conceptual scenario applies blockchain technology to strengthen data integrity and decrease data transmission risk
	[83]	C3, C4	The proposed Blockchain-based Shared Manufacturing (BSM) framework is applied to support Cyber Physical Systems (CPS)
	[61]	C2, C4	The proposed ManuChain takes advantage of blockchain-driven smart contracts to proactively decentralize task execution and make the results available for optimization
	[84]	C1, C2	The proposed smart manufacturing conceptual scenario applies blockchain technology to strengthen data integrity and decrease data transmission risk
